# Cholinesterase inhibitors-associated torsade de pointes/QT prolongation: a real-world pharmacovigilance study

**DOI:** 10.3389/fphar.2023.1343650

**Published:** 2024-01-11

**Authors:** Ni Zhang, Lanlan Gan, Guiyuan Xiang, Jing Xu, Tingting Jiang, Yanping Li, Yuanlin Wu, Rui Ni, Yao Liu

**Affiliations:** Department of Pharmacy, Daping Hospital, Army Medical University, Chongqing, China

**Keywords:** cholinesterase inhibitors, torsade de pointes/QT prolongation, pharmacovigilance study, drug safety, FAERS

## Abstract

**Objective:** Cholinesterase inhibitor (ChEIs) is the first-line drug for Alzheimer’s disease (AD). Understanding torsade de pointes (TdP)/QT prolongation with different ChEIs is essential for its safe and rational administration. This study aimed to evaluate the correlation between different ChEIs and TdP/QT prolongation.

**Methods:** All ChEIs related TdP/QT prolongation cases were retrieved from the FAERS database using standard MedDRA query (SMQ) from the first quarter of 2004 to the third quarter of 2022. Disproportionality and sensitivity analysis were used to determine the signal of TdP/QT prolongation related to ChEIs.

**Results:** 557 cases of TdP/QT prolongation related to 3 ChEIs were searched by SMQ. The patients were mostly elderly people, with markedly more female than male. The signals of TdP/QT prolongation for ChEIs were detected by disproportionality analysis, and the signal of Donepezil was the strongest. The sensitivity analysis results indicate a robust and stable correlation between these signals with ChEIs. TdP/QT prolongation usually occurs within 1 month after taking ChEIs. The drug with the highest frequency of combination with donepezil and galantamine is citalopram, and the drug with the highest frequency of combination with rivastigmine is atorvastatin.

**Conclusion:** The signals of TdP/QT prolongation related to ChEIs were strong and stable. It is necessary to be vigilant about the TdP/QT prolongation of various ChEIs, especially in elderly women, the initial stage after taking ChEIs, and when ChEIs combining with drugs that could prolong the QT interval.

## 1 Introduction

Alzheimer’s disease (AD) is one of the most common age-related neurodegenerative diseases. It is the most common form of dementia. Its characteristics include progressive cognitive impairment, various behaviors that seriously interfere with daily life, and neuropsychiatric disorders (Daly et al., 2001; Khairallah and Kassem, 2011). With the aging population and the increasing number of patients, the treatment of AD has become increasingly important. Cholinesterase inhibitors (ChEIs), such as donepezil, galantamine and rivastigmine, are commonly used in clinical treatment of AD (Hogan, 2014). As a first-line drug for AD, ChEIs can increase cholinergic transmission through reversible and noncompetitive inhibition of synaptic cholinesterase, thereby improving cognitive function, neuropsychiatric symptoms, and ability of daily living in mild and moderate patients (Jann, 1998; Birks and Grimley Evans, 2015).

With the wide application of ChEIs in AD patients, cardiovascular adverse events related to ChEIs have attracted more and more attention (Howes, 2014). Several studies have reported the case of Torsade de pointes (TdP)/QT prolongation during ChEIs therapy (Leitch et al., 2007; Fisher and Davis, 2008; Tanaka et al., 2009). In addition, Health Canada also strengthened the information related to the TdP/QT prolongation of ChEIs(canada, 2022). Since QT prolongation may induce TdP and cause sudden cardiac death, the characteristics of TdP/QT prolongation related to different ChEIs for give them proper and safe administration were essential.

Currently, the safety evidence of the three ChEIs is still insufficient, and long-term monitoring is needed. The spontaneous reporting system database is the primary source of data for detecting signals of adverse reactions, and some rare adverse reactions have been identified through this way (Montastruc et al., 2016). This retrospective, pharmacovigilance study aims to survey the relationship and characteristics between three ChEIs with TdP/QT prolongation through FAERS database.

## 2 Methods

### 2.1 Data source and study design

A disproportionality, pharmacovigilance, retrospective study was conducted from the FAERS database to evaluate the risk of TdP/QT prolongation from various ChEIs in a large-scale population. FAERS is the primary information source used by FDA for post marketing safety monitoring and evaluation, and has collected adverse event reports from physicians, pharmacists, pharmaceutical enterprises, consumers all over the world (Zhao et al., 2023). All adverse events in FAERS were coded with preferred terms (PT) of Medical Dictionary for Drug Regulatory Activities (MedDRA). We analyzed the adverse event reports of ChEIs during the period from January 2004 to September 2022.

### 2.2 Adverse event definition

Donepezil, galantamine and rivastigmine were enrolled in this study. The definition of Torsade de pointes/QT prolongation is based on the standard MedDRA query (SMQ), including 6 PT such as Electrocardiogram QT interval abnormal (MedDRA code 10063748), Electrocardiogram QT prolonged (MedDRA code 10014387), Long QT syndrome (MedDRA code 10024803), Long QT syndrome congenital (MedDRA code 10057926), Torsade de pointes (MedDRA code 10044066), Ventricular tachycardia (MedDRA code 10047302).

ChEIs, including donepezil, galantamine and rivastigmine, were identified to obtain report data from the FAERS. We search each drug by generic and brand names: generic name “donepezil”, “galantamine”, “rivastigmine” and brand name “Aricept”, “Adlarity”, “Razadyne”, “Reminyl”, “Exelon”. The demographics and clinical characteristics of patients, such as age, sex, outcome, reporters’ type, country, start-dt and event-dt were collected. We collected and organized the top 30 drugs in terms of frequency of concomitant medication with ChEIs to analyze the influence of concomitant medication. For comprehensive analysis, ChEIs with >3 reported TdP/QT prolongation cases were included in the study.

### 2.3 Statistical analysis

Disproportionality analysis is a commonly employed approach for detecting signals of adverse events in the world. The following data-mining algorithms were used for disproportionality analysis and signal detection: Reporting Odds Ratio (ROR), Proportional Reporting Ratio (PRR) and Information Content (IC) (Sakaeda et al., 2013). Data mining algorithms are statistical tools that can detect signals indicating adverse events associated with drugs. Currently, these algorithms are employed by Medicine and Healthcare Products Regulatory Agency (MHRA), UK, Netherlands pharmacovigilance Center, World health Organization (WHO), and FDA (Sakaeda et al., 2013). These algorithms are used to calculate signal scores to ascertain a significant association between a drug and an adverse event of interest.

## 3 Results

### 3.1 Clinical characteristics of TdP/QT prolongation cases related to ChEIs

From January 2004 to September 2022, a total of 33,626 cases of TdP/QT prolongation -related reports were found in the FAERS database. A total of 557 cases of TdP/QT prolongation associated with ChEIs were identified. Of these, a total of 430 cases were found in reports pertaining to Donepezil, 54 for Galantamine, and 73 for Rivastigmine.

The characteristics of TdP/QT prolongation-related reports submitted for ChEIs were described in Table 1. The gender subset analysis showed that ChEIs associated with TdP/QT prolongation reports were higher in female patients (63.73%) than male patients (21.54%). More cases of ChEIs related TdP/QT prolongation were found in the patient group of over 75 years old. Patients exposed to ChEIs were mainly from United Kingdom, Japan, and United States. Most cases were reported following 2012.

**TABLE 1:** The clinical information of cases with TdP/QT prolongation related to ChEIs.

Characteristics	Donepezil	Galantamine	Rivastigmine	Total
Gender				
Male	83	16	21	120
Female	281	27	47	355
Unkown	66	11	5	82
Age(years old)				
<18	0	0	0	0
18–44	0	0	0	0
45–64	3	0	1	4
65–74	40	3	7	50
≥75	304	33	42	379
unknown	83	18	23	124
Reporting region				
United Kingdom	175	7	9	191
Japan	91	18	13	122
United States	55	5	7	67
other	80	24	37	141
unknown or missing	29	0	7	36
Report year				
2004	6	1	4	11
2005	10	0	3	13
2006	7	2	3	12
2007	15	1	4	20
2008	16	0	6	22
2009	19	6	2	27
2010	11	1	6	18
2011	11	1	3	15
2012	26	2	10	38
2013	24	1	1	26
2014	10	5	0	15
2015	54	3	2	59
2016	24	5	3	32
2017	43	9	5	57
2018	37	4	6	47
2019	44	7	3	54
2020	40	4	6	50
2021	22	2	2	26
2022	11	0	4	15

The outcomes of TdP/QT prolongation induced by ChEIs were summarized in Table 2, mainly involving serious outcomes. In total, TdP/QT prolongation related to CHEIs was accompanied by hospitalizations in 268 (48.11%) cases.

**TABLE 2:** The percent of outcome of each ChEIs induced to TdP/QT prolongation.

Outcome	Number of outcomes (%)			
	Donepezil	Galantamine	Rivastigmine	Total
Death	9(2.09)	1(1.85)	11(15.07)	21(3.77)
Disability	0(0)	0(0)	0(0)	0(0)
Hospitalization-initial or prolonged	231(53.72)	15(27.78)	22(30.14)	268(48.11)
Life-threatening	95(22.09)	8(14.81)	5(6.85)	108(19.39)
Required intervention to prevent permanent impairment/damage	2(0.47)	0(0)	0(0)	2(0.36)
Other	93(21.63)	30(55.56)	35(47.94)	158(28.37)
Total	430(100.00)	54(100.00)	73(100.00)	557(100.00)

We have compiled high-frequency drugs used in combination with ChEIs, including the top 30 drugs with the highest frequency of combination with donepezil, galantamine and rivastigmine, as shown in Table 3. The drug with the highest frequency of combination with donepezil and galantamine is citalopram, and the drug with the highest frequency of combination with rivastigmine is atorvastatin.

**TABLE 3:** Concomitant medication and frequency of ChEIs.

Suspected drug	Concomitant medications and frequency
Donepezil	citalopram(78), simvastatin(73), aspirin(62), omeprazole(61), fluoxetine(53), bendroflumethiazide(39), acetaminophen(34), atorvastatin(33), gabapentin(29), calcium(28), hydroxychloroquine(26), perindopril(26), alendronate alendronic acid(24), metoclopramide(24), amlodipine(23), levothyroxine(21), bisoprolol(21), lansoprazole(20), quinine(20), furosemide(19), memantine(18), diltiazem(17), clopidogrel(16), sertraline(16),bumetanide(16), pravastatin(16), propofol(15), mirtazapine(14), zolpidem(14), amiodarone(14)
Galantamine	citalopram(19), irbesartan(10),acetaminophen(9), atorvastatin(8), amlodipine(7), furosemide(6), indapamide(6), levothyroxine(5), allopurinol(4), bisoprolol(4),captopril(4), flecainide(4), hydrochlorothiazide(4), nifedipine(4), risperidone(4), sertraline(4), warfarin(4), aspirin(3), amiodarone(3), calcium(3), cilostazol(3), losartan(3), olanzapine(3), omeprazole(3), quetiapine(3), spironolactone(3), aliskiren(3), haloperidol(2), levofloxacin(2), domperidone(2)
Rivastigmine	atorvastatin(11), aspirin(10), olanzapine(8), risperidone(8), carbidopa levodopa(6), citalopram(6), levothyroxine(6), amisulpride(4), memantine(4), memantine(4), amlodipine(3), nilotinib(3), propafenone(3), amitriptyline(2), bisoprolol(2), clopidogrel(2), duloxetine(2), haloperidol(2), metformin(2), montelukast(2), nitroglycerin(2), omeprazole(2), propranolol(2), quetiapine(2), rivaroxaban(2), sertraline(2), simvastatin(2),furosemide(2), calcium carbonate(2), lidocaine(2)

Note: Only the top 30 frequency ranked concomitant medications were recorded.

### 3.2 Disproportionality analysis

The results of data-mining algorithms were summarized in Table 4. Overall, the values of all 3 methods indicated that ChEIs was correlated with TdP/QT prolongation. In terms of the criteria of signal detection, donepezil demonstrated the strongest potential correlation to TdP/QT prolongation, with the values of ROR = 8.98, 95% CI = (8.16, 9.89); PRR = 8.88, χ^2^ = 2944.95 and IC = 3.09, IC025 = 2.95.

**TABLE 4:** Comparison of TdP/QT prolongation signals related to different ChEIs.

	N	ROR	PRR	IC
		(95%CI)	(χ^2^)	(IC025)
Donepezil	430	8.98(8.16,9.89)	8.88(2944.95)	3.09(2.95)
Galantamine	54	5.12(3.92,6.68)	5.11(175.44)	2.24(1.86)
Rivastigmine	73	1.68(1.34,2.12)	1.68(19.38)	0.73(0.40)

Note: criteria of positive signals: ROR, 95%CI > 1, N ≥ 3; PRR, PRR ≥ 2, χ^2^ ≥ 4; IC, IC025 > 0.

N, number; ROR, reporting odds ratio; CI, confidence interval; PRR, proportional reporting ratio; χ^2^, chi-square; IC, information component; IC025, the lower limit of the 95% two-sided CI of the IC.

### 3.3 Sensitivity analysis

**FIGURE 1:**
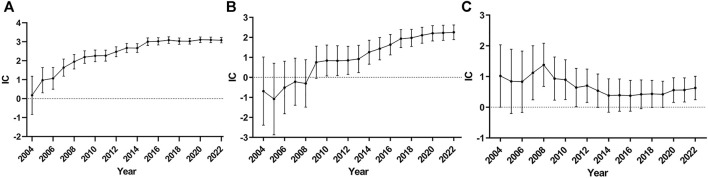
Time scan of IC for ChEIs associated TdP/QT prolongation. **(A)** donepezil related-TdP/QT prolongation; **(B)** galantamine related-TdP/QT prolongation; **(C)** rivastigmine related-TdP/QT prolongation.

To reduce false positive signals related to TdP/QT prolongation of ChEIs, it is necessary to demonstrate that the information component (IC) and 95% confidence interval (CI) are stable over time, so we drew the diagram of time scan respectively. When the time scan is steady upward trend and the 95%CI narrowed, the signal is stable and strong association (Orre et al., 2000). The results are shown in Figure 1, of which Figures 1A shows the donepezil related-TdP/QT prolongation, Figures 1B for galantamine, and Figures 1C for rivastigmine.

From Figures 1A, we can see the value of IC increased markedly and the lower 95% CI limit above zero in 2005 as the number of reports of donepezil associated TdP/QT prolongation increased, which indicates the signal first appeared in 2005. From 2005 to 2022, the interval of the IC becomes smaller, and it became stable in 2014, which means a stable signal and strong association. The time scan of galantamine-associated TdP/QT prolongation and rivastigmine-associated TdP/QT prolongation was shown in Figures 1B, C. In 2010, galantamine first appeared the signal of TdP/QT prolongation. In the following 12 years, the 95% CI gradually narrowed and showed an upward trend, indicating that the signal was stable. For the rivastigmine-associated TdP/QT prolongation, the first signals of rivastigmine appeared in 2007. From 2007 to 2022, the interval of IC becomes smaller, which indicates a trend to a high association and stable signal. These changes could largely eliminate the possibility that signals of ChEIs-associated TdP/QT prolongation were false positives.

### 3.4 Events onset time

**FIGURE 2:**
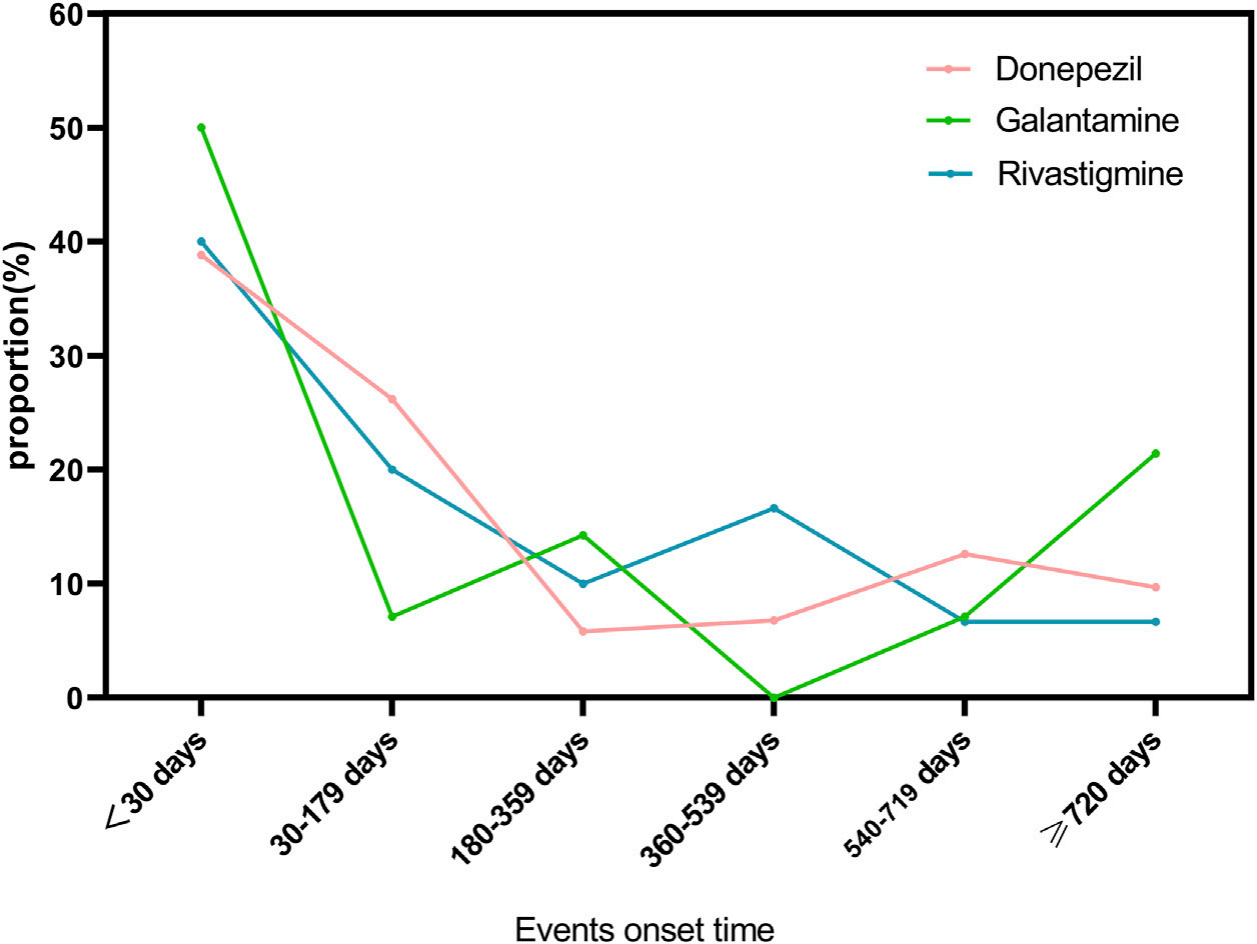
Events onset time for ChEIs associated TdP/QT prolongation.

As shown in Figure 2, we have analyzed the onset time of TdP/QT prolongation caused by ChEIs. It is worth noting that TdP/QT prolongation mostly occur within 1 month after taking ChEIs. Generally, the median adverse event onset interval for Donepezil was 52(IQR5-539) days, 48.5(IQR7-695.75) days for Galantamine, and 82(IQR8-420.75) days for Rivastigmine.

## 4 Discussion

A clinical trial involving 162 patients with AD demonstrated that electrocardiogram (ECG) parameters remained unchanged following treatment with three ChEIs when compared to the initial baseline (Isik et al., 2012). Consequently, our pharmacovigilance studies are of great significance in terms of monitoring drugs after they enter the market. More general or rare adverse reactions to ChEIs may only be uncovered when these drugs are exposed to a larger population. We analyzed reports from the FAERS database to examine the features of cases involving TdP/QT prolongation associated with donepezil, galantamine, and rivastigmine. Meanwhile, the signal value of TdP/QT prolongation caused by three ChEIs and the associated clinical outcomes were compared. Based on the evaluation of ROR, PRR, and IC, the findings demonstrate a significant and stable signal association between ChEIs and TdP/QT prolongation.

Due to the growing use and comprehension of ChEIs, the potential hazard of TdP/QT prolongation induced by these medications has gained greater significance (Takaya et al., 2009; Malik, 2019). Although cardiovascular issues of these medications are less frequent than gastrointestinal, they still need to be considered in susceptible individuals. Safety reviews conducted by Health Canada and previous studies (Walsh and Dourish, 2002; Fisher and Davis, 2008; Kuwahata et al., 2021) suggest that the risks associated with ChEIs and their potential to cause QT prolongation may be underestimated. The findings of this examination revealed that a larger proportion of individuals aged over 75 years and of female gender experienced TdP/QT prolongation following the consumption of ChEIs. The incidence and prevalence of AD increases with age, making the 75 plus elderly at increased chances of receiving the ChEIs. This will translate to higher TdP/QT prolongation reports coming from this population. Generally, the risk of QT prolongation is considered higher in women, and studies have reported the risk of antipsychotic drugs on QT interval prolongation in women (Suzuki et al., 2013). A large cross-sectional registry study in Sweden showed that in the elderly population with severe neurocognitive impairment, the use of drugs that prolong the QT interval was significantly associated with factor in women (Gustafsson et al., 2023). Studies have shown that females are at increased risk for QT prolongation compared to males, likely due to their longer baseline QT intervals and gender-specific differences in cardiac delayed rectifier potassium channel expression regulated by estrogen (Rabkin, 2015). In addition, our study found that among the top 30 drugs used in combination with ChEIs, there are drugs such as citalopram, fluoxetine, sertraline, mirtazapine, risperidone, quetiapine, olanzapine and amiodarone that can prolong the QT interval (Tisdale, 2016). Zeltser’s research (Zeltser et al., 2003) indicated that in addition to the risk factor of women, other risk factors include structural heart disease, hypokalemia, and overdose, and the combination of other medications that can cause QT prolongation. Our research relies on spontaneous reports, limiting our access to information regarding patient medical history and additional conditions. It remains uncertain whether there exist other risk factors for TdP/QT prolongation, and additional research is necessary to evaluate. However, elderly women taking ChEIs and those using drugs that can prolong the QT interval should pay extra attention to TdP/QT prolongation based on current research findings.

CredibleMeds maintains a comprehensive list of medications associated with QT interval prolongation and arrhythmia over time. According to the association between drugs and TdP/QT prolongation, the website classifies drugs into four different risk categories (Woosley et al., 2023). Donepezil was classified as a known risk in 2015, Galantamine was placed on the conditional risk list, and Rivastigmine was not in the four lists. However, this study identified that all three medications showed signals of TdP/QT prolongation based on disproportionality analysis. Several studies indicate that ChEIs inhibits acetylcholinesterase, leading to increased transmission of this neurotransmitter to increase the cholinergic levels in the brain. The voltage-gated calcium channel is activated through the activation of cardiac receptors. Elevated intracellular calcium levels cause an increase in phase 2 of the cardiac action potential cycle, ultimately raising the likelihood of ventricular arrhythmias (Malik et al., 2019). Additionally, ChEIs have been proven to be effective blockers of hERG channels, which are the basis for the rapid activation of delayed rectifier currents. These currents play an important role in determining the repolarization of the action potential in ventricular myocytes. Abnormal functions in the hERG channel can result in QT prolongation, which can lead to TdP (Vigneault et al., 2012b; Chae et al., 2015). Lastly, it is important to consider both the expected effectiveness and the possible safety concerns when choosing medications for managing AD in individual patients. Therefore, we recommend that physicians exercise caution when prescribing ChEIs, particularly for patients with underlying heart conditions or who are taking medications known to cause QT prolongation (Jackson and Stowe, 2019).

The three ChEIs related TdP/QT prolongation signals are arranged in order as donepezil [ROR = 8.98,95%CI (8.16,9.89); PRR = 8.88, χ^2^ = 2944.95; IC = 3.09, IC025 = 2.95], galantamine [ROR = 5.12,95%CI (3.92,6.68); PRR = 5.11, χ^2^ = 175.44; IC = 2.24, IC025 = 1.86] and rivastigmine [ROR = 1.68,95%CI (1.34,2.12; PRR = 1.68, χ^2^ = 19.38; IC = 0.73, IC025 = 0.40)]. The signal differences caused by these three drugs seem to be explained by the plasma concentration of the drugs (due to either physiologically clearance of ChEIs or inhibition of its metabolism by other drugs). Donepezil has a half-life of 70 h, galantamine has 7 h, rivastigmine has 1.5 h (Rubey, 2003). The large difference in half-lives leads to different amounts of accumulation in the body, which may result in different signals. In addition, donepezil and galantamine are mainly metabolised by cytochrome P450 (CYP) 2D6 and CYP3A4 in the liver, rivastigmine is metabolised by sulfate conjugation (Noetzli and Eap, 2013). CYP2D6 poor metabolizers (∼7% of Caucasians) have shown a 25% decrease in clearance and a 35% increase of area under the curve (AUC_∞_) of unchanged galantamine, compared to extensive metabolizers (Vigneault et al., 2012a). Compared with the CYP2D6 extensive metabolizers, poor metabolizers had a 32% slower clearance of donepezil (Noetzli et al., 2014). However, the nonhepatic metabolism route of rivastigmine limits its pharmacokinetic interactions with other medications (Rubey, 2003). The influence of different genotypes on drug clearance rate is also one of the reasons why we explain the different signals.

To our knowledge, few studies have analyzed the onset time of TdP/QT prolongation caused by ChEIs. A retrospective study examined the potential correlation between long-term treatment with Donepezil and changes in ECG. The study found a significant association between the use of Donepezil for over a year and prolongation of the PR, QRS, and QT intervals (Kho et al., 2021). It should be noted that this study has a retrospective design with a primary focus on individuals of Caucasian descent and did not analyze and compare data from less than a year. We conducted an onset time analysis, which indicated a need for heightened awareness of TdP/QT prolongation associated with ChEIs, particularly during the initiation phase of treatment.

Unavoidably, our research still has limitations. Firstly, this study did not consider the specific dosage, and other factors. Secondly, due to the inherent limitations of the spontaneous reporting system, we were unable to collect specific laboratory test values, co-morbid conditions of patients and clinical symptoms from patients, resulting in biases and limitations in the results. Furthermore, signal detection merely suggests the possibility of risks, and definitive causal relationships require further research and evaluation to determine. Finally, as FAERS relies on spontaneous reporting data, there may be considerable gaps or uncertainties in the information, which could potentially affect the outcomes.

## 5 Conclusion

Concerns about safety issues, especially in TdP/QT prolongation have arisen due to widespread use of ChEIs. Although the FAERS database has limitations, it can successfully detect the TdP/QT prolongation associated with ChEIs and provide important clinical characteristics. Our pharmacovigilance analysis prompted clinicians to raise concerns about potential TdP/QT prolongation adverse events that elderly women taking ChEIs. Additionally, there are drugs more commonly associated with TdP/QT prolongation such as fluoroquinolones, amiodarone, antipsychotics and antidepressants and which are commonly presecribed in AD patients at some point for inevitable reasons. Consequently, caution should be particularly followed in AD patients prescribed ChEIs with these drug classes.

## Data Availability

The raw data supporting the conclusion of this article will be made available by the authors, without undue reservation.
